# Popliteal artery aneurysm in a 14-year-old boy with tuberous sclerosis complex

**DOI:** 10.1016/j.jvscit.2022.06.016

**Published:** 2022-07-16

**Authors:** Ryohei Maeno, Toshihiko Isaji, Toshio Takayama, Katsuyuki Hoshina

**Affiliations:** Division of Vascular Surgery, Department of Surgery, Graduate School of Medicine, The University of Tokyo, Tokyo, Japan

**Keywords:** Aneurysm, Popliteal artery, Tuberous sclerosis complex

## Abstract

Tuberous sclerosis complex (TSC) is rarely associated with aneurysms. We have described a patient with a popliteal artery aneurysm that was associated with TSC and occlusion of the right posterior tibial artery. The patient underwent aneurysm resection and vein graft replacement, with an uneventful postoperative course and no recurrence at 11 months of follow-up. Patients with TSC could have aneurysms in areas that will not be recognized on abdominal imaging. Physical examination of the lower extremities should be performed owing to the possibility of a popliteal artery aneurysm, and imaging studies should be performed if an aneurysm is suspected.

Aneurysms are notably rare in children and are often associated with connective tissue diseases such as Marfan syndrome and Ehlers-Danlos syndrome. They can also be caused by autoimmune or infectious vasculitis (eg, Takayasu disease and Kawasaki disease), popliteal artery syndromes, trauma, and tuberous sclerosis complex (TSC).^1^ TSC is inherited in an autosomal dominant manner and was first reported by von Recklinghausen^2^ in 1862 and named by Bourneville^3^ in 1880. The typical triad of symptoms is epilepsy, mental retardation, and facial angiofibromas; however, only a few individuals will develop all these signs. The symptoms are systemic, and various associated lesions have been documented in the brain, heart, lungs, kidneys, spleen, liver, uterus, and soft tissues.^4^ TSC is caused by mutations in either the *TSC1* or *TSC2* gene, encoding intracellular proteins, hamartin, or tuberin. Hamartin and tuberin act as endogenous regulators of the mechanistic targets of rapamycin activity (mTOR), a signaling pathway involved in cell proliferation, nutrient uptake, and transcriptional and translation control. Mutations in TSC1 and TSC2 are thought to hyperactivate the mTOR pathway and cause various symptoms.^5^ Complications with arterial aneurysms are notably rare in those with TSC, and the mechanism of pathogenesis has long remained unclear. However, it has recently been hypothesized that the characteristic changes in smooth muscle cells due to mTOR hyperactivation might be responsible for this.^5^

Although aneurysms associated with TSC have been reported, studies of popliteal artery aneurysms (PAAs) are scarce. We have described a patient with TSC and a PAA and reviewed the literature on aneurysms associated with TSC. The patient and his parent provided written informed consent for the report of his case details and imaging studies.

## Case report

A 14-year-old boy had fallen during a long-distance run and had presented to his local orthopedic clinic with right knee pain. He was suspected of having a meniscus injury and underwent magnetic resonance imaging (MRI) at the same hospital, which incidentally showed a right PAA. He was referred to our hospital. The patient had a history of cardiac rhabdomyoma, cortical nodules, subepicardial nodules (noted since the neonatal period), lobular leukoplakia (at 4 months old), epilepsy (at 5 months old), shagreen patches (at 5 years old), and facial angiokeratomas (at 12 years old). No genetic diagnosis was performed; however, a definitive diagnosis of TSC was made. Routine head MRI, abdominal MRI, and echocardiography were performed at the pediatric department of our hospital for the surveillance of the TSC-associated diseases. His mother had had TSC and had died of glioblastoma, which is frequently associated with TSC.

**Fig 1:**
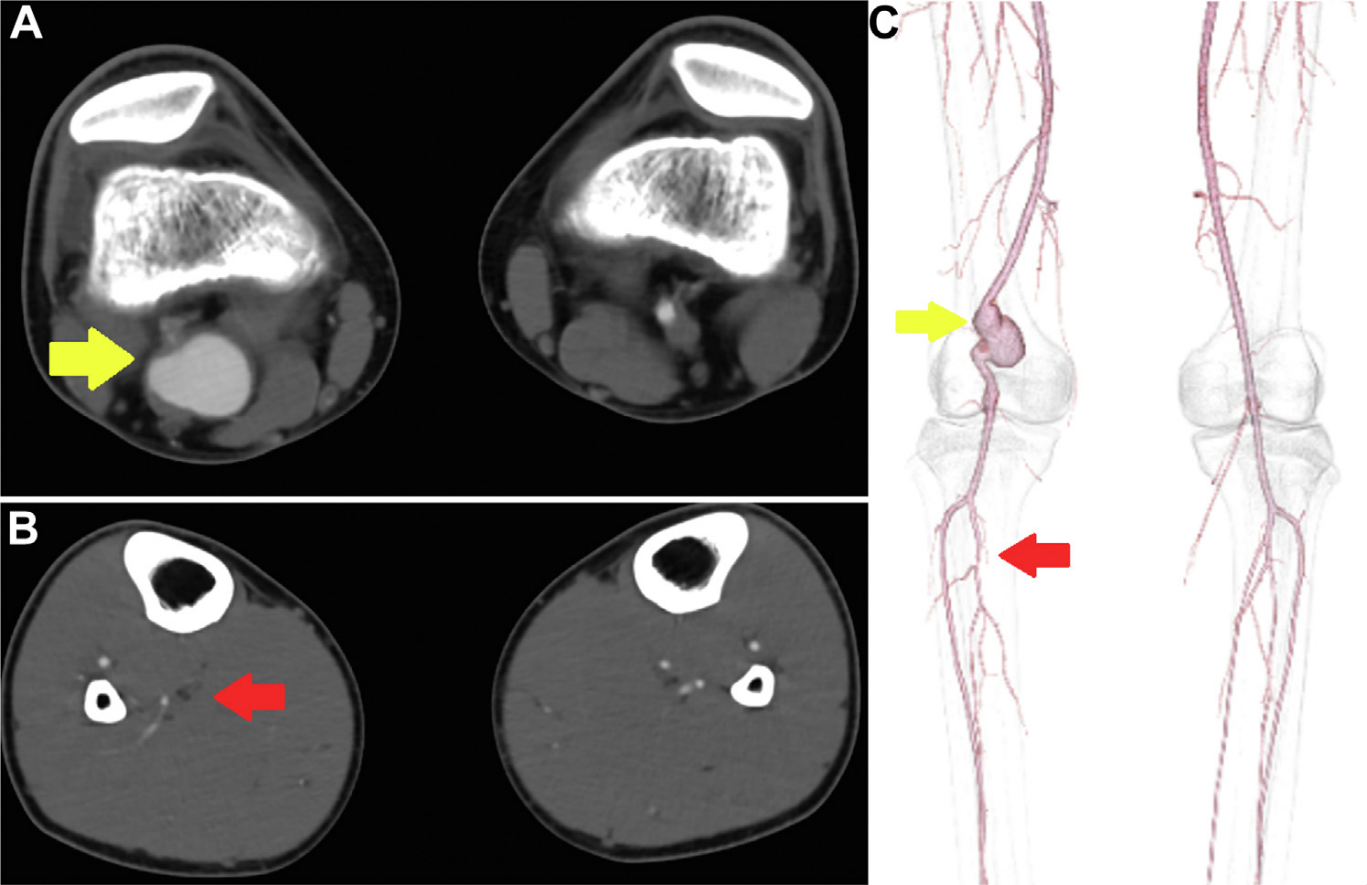
Preoperative computed tomography (CT) findings. **A,** Right popliteal aneurysm (*yellow arrow*) 47 × 33 × 27 mm in size. **B,** Occlusion of the right posterior tibial artery (*red arrow*). **C,** Three-dimensional computed tomography angiogram of the lower limb artery.

On physical examination, a pulsatile mass was palpable in the right popliteal artery, and the right posterior tibial artery had a weak pulse. The ankle brachial index was 0.93 on the right side and 1.05 on the left side. Contrast-enhanced computed tomography (CT) revealed a right PAA sized 47 × 33 × 27 mm and an occluded right posterior tibial artery (Fig 1).

**Fig 2:**
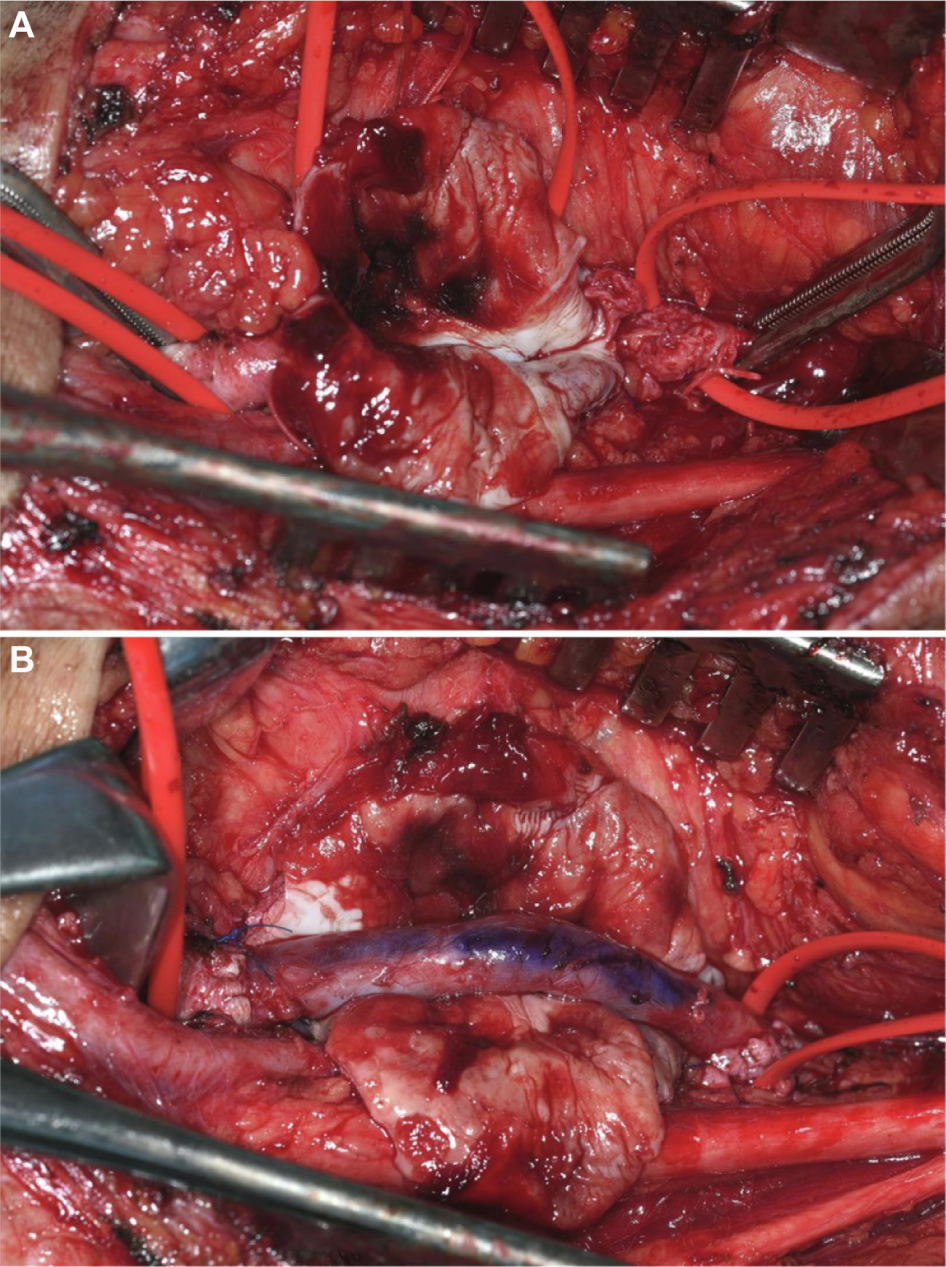
Intraoperative findings. **A,** The popliteal artery aneurysm (PAA) was exposed and incised. **B,** Replacement with a reversed vein graft by end-to-end anastomoses.

Aneurysm resection and vein graft replacement via the posterior approach for the right PAA were performed with the patient in the prone position (Fig 2). A reversed ipsilateral great saphenous vein was used as the graft. The proximal and distal end-to-end anastomoses of the artery and graft were formed at an angle using a continuous suture with a little extra length of the graft.

The aneurysm was saccular with a small intramural thrombus, and its wall was soft and elastic. Histologic evaluation showed thickening of the entire wall, a small amount of tunica media consisting of elastic fibers, unclear atherosclerosis, and calcification, poor inflammatory cell infiltration, and no granuloma. These findings were similar to the histologic findings of aortic aneurysms associated with TSC.^5^^,^^6^ After confirming graft patency with a duplex ultrasound scan on postoperative day 4, the patient was discharged on postoperative day 7. Postoperative genetic testing showed no evidence of any connective tissue disorders, including Marfan syndrome and Ehlers-Danlos syndrome. At 11 months postoperatively, the patient had not developed new lesions, recurrence of graft occlusion, or aneurysm recurrence.

## Discussion

TSC is an autosomal dominant disease that affects various organs and is rarely associated with aneurysms.^4^ A search of PubMed for “aneurysm” and “tuberous sclerosis complex” revealed 84 cases of aneurysms associated with TSC (Table I).^5^, ^6^, ^7^, ^8^, ^9^, ^10^, ^11^, ^12^, ^13^, ^14^, ^15^, ^16^, ^17^, ^18^, ^19^, ^20^, ^21^, ^22^, ^23^, ^24^, ^25^, ^26^ Studies have reported aneurysms in the intracranial artery, aorta, carotid artery, pulmonary artery, axillary artery, subclavian artery, and iliofemoral artery, with intracranial and aortic aneurysms the most common.

**Table I:** Reported cases of aneurysms associated with tuberous sclerosis complex (TSC)

Anatomic location (references)	Aneurysms, No.
Intracranial artery (6)	42
Aorta (5, 7-17)	32
Carotid artery (18-20)	3
Pulmonary artery (21-23)	3
Axillary artery (24, 25)	2
Subclavian artery (26)	1
Iliofemoral artery (1)	1

The location and number of aortic aneurysms associated with TSC are listed in Table II. Aortic aneurysms associated with TSC are usually detected in young patients and are known to have a high risk of rupture owing to the rapid increase in the aneurysm diameter.^27^ Open surgery should be performed as soon as possible after the diagnosis, regardless of the diameter.^16^ Because most aortic aneurysms will remain undetected until rupture or a postmortem examination, surveillance for aneurysms with imaging modalities, such as abdominal ultrasound, for patients with TSC is crucial.^28^ Surveillance for new lesions and complications via abdominal ultrasound or CT or MRI is recommended every 2 to 3 years until puberty and annually thereafter.^1^ For TSC patients with intracranial aneurysms, the proportion of large or giant intracranial aneurysms has been reported to be higher in younger patients, suggesting the rapid growth of these lesions in pediatric TSC patients. Therefore, regular MRI screening has been recommended to promptly identify the growth of intracranial aneurysms.^7^

**Table II:** Reported cases of aortic aneurysms associated with tuberous sclerosis

Investigator	Aneurysms, No.	Age	Anatomic aneurysm location	Outcome
Salerno et al,^8^ 2010^a^	21	6 months to 43 years (median, 2.5 years)	Abdominal, 15; thoracic, 4; abdominal and thoracic, 2	Repaired, 15; ruptured, 4; repaired (died after repair), 2
Towbin et al,^9^ 1987	1	9 months	Abdominal	Repaired (died after repair)
Bavdekar et al,^10^ 2000	1	6 years	Thoracoabdominal	Died of respiratory failure
Bahena et al,^11^ 2005	1	8 months	Abdominal	Died of aortic dissection
Ye et al,^12^ 2012	1	17 months	Abdominal	Repaired
Bailey et al,^13^ 2013	1	5 years	Thoracoabdominal	Repaired
Sawan et al,^14^ 2015	1	5 years	Thoracoabdominal	Repaired
Eliason et al,^15^ 2016	1	5 years	Abdominal and thoracic	Repaired
Dueppers et al,^16^ 2017	1	9 years	Abdominal	Repaired
Geiger et al,^17^ 2019	1	26 years	Thoracic	Repaired
Hedin et al,^5^ 2021	1	2 years	Abdominal	Repaired
Byrne et al,^6^ 2021	1	9 years	Thoracic	Repaired

a Study period, 1971 to 2010.

PAA is another disease that requires early resection to prevent thromboembolic events and rupture. True PAAs in the pediatric population, such as in the present case, rarely disappear spontaneously and will usually be detected because of symptoms of lower extremity ischemia due to thromboembolism.^29^ Our patient did not report ischemic symptoms, and the PAA had been incidentally detected by screening of the lower leg. However, screening with imaging modalities is usually performed of the abdomen or chest; thus, lesions elsewhere could be overlooked. TSC can form aneurysms in a variety of locations, and the presence of aneurysms in the carotid, axillary, subclavian, and lower extremity arteries can be evaluated by physical examination and palpation of a mass. A careful physical examination is important because the aneurysms associated with TSC can have fatal complications.

## Conclusions

We have reported the case of a PAA in a 14-year-old boy with TSC that had been incidentally detected by MRI performed for a knee injury. Surgical bypass was successfully performed for this patient, and no complications or new lesions were detected. Because TSC can cause symptoms in various parts of the body, surveillance with imaging modalities, including CT or MRI, and physical examination are critical.
